# A multi-center psychometric evaluation of the Severity Indices of Personality Problems 118 (SIPP-118): Do we really need all those facets?

**DOI:** 10.1007/s11136-020-02654-8

**Published:** 2020-10-07

**Authors:** Muirne C. S. Paap, Benjamin Hummelen, Johan Braeken, Espen A. Arnevik, Espen Walderhaug, Theresa Wilberg, Han Berghuis, Joost Hutsebaut, Geir Pedersen

**Affiliations:** 1 Department of Research and Innovation, Division of Mental Health and Addiction, Oslo University Hospital, P.O.Box 4956, 0424 Nydalen, Oslo Norway; 2 Department of Child and Family Welfare, Faculty of Behavioural and Social Sciences, University of Groningen, Groningen, The Netherlands; 3 Centre for Educational Measurement at the University of Oslo (CEMO), University of Oslo, Oslo, Norway; 4 Department of Addiction Treatment, Division of Mental Health and Addiction, Oslo University Hospital, Oslo, Norway; 5 Institute of Clinical Medicine, University of Oslo, Oslo, Norway; 6 ARKIN Mental Health, NPI Centre for Personality Disorders, Amersfoort, The Netherlands; 7 Viersprong Institute for Studies on Personality Disorders (VISPD), Halsteren, The Netherlands; 8 Department of Personality Psychiatry, Division of Mental Health and Addiction, Oslo University Hospital, Oslo, Norway; 9 NORMENT, KG Jebsen Center for Psychosis Research, Institute of Clinical Medicine, University of Oslo, Oslo, Norway

**Keywords:** Personality disorders, SIPP-118, Personality traits, Subscales, Distinctiveness, PRMSE, Value-added ratio, Multi-center study

## Abstract

**Purpose:**

The Severity Indices of Personality Problems 118 (SIPP-118) is a self-report questionnaire that aims to measure core components of (mal)adaptive personality functioning that can change over time. In this study, we aimed to assess the facet strength of the 16 facets across three large clinical samples.

**Methods:**

Data from Norwegian and Dutch psychiatric patients were analyzed in this international multi-center study (N_1_ = 2814, N_2_ = 4751, N_3_ = 2217). Bi-factor modeling was used to assess to what degree the SIPP items tap into an overall general factor. The incremental value (distinctiveness) of the facets was studied using proportional reduction in mean squared error (PRMSE) based statistics.

**Results:**

The estimated model showed adequate fit. The explained common variance (ECV) attributable to the general factor equaled 50% for all three samples. All but two facets (stable self-image and frustration tolerance) showed sufficient levels of distinctiveness. The findings were observed to be comparable across the three samples.

**Conclusion:**

Our findings showed that the general factor was relatively weak, and the facets had a clear incremental value.

## Introduction

Screening for personality pathology is of paramount importance; especially in clinical settings. Studies have shown that between 3 and 10% of the general population meet the diagnostic criteria of one or more personality disorders [1, 2]. Prevalence rates in psychiatric populations have been found to be substantially higher: 45–51% in US samples and 40–92% in European samples [3]. Personality disorders are characterized by considerable suffering and/or lasting impairment of social adaptiveness. Patients diagnosed with personality disorders have a higher risk for suicide, and often suffer from psychosocial impairment, experience decreased work capacity and have inadequate skills for establishing lasting interpersonal relationships [4].

Traditionally, personality traits (including maladaptive ones) have been regarded as stable. However, there is a growing body of research that focuses on and finds support for changeable aspects of personality. In general psychology as well as in psychiatry, a distinction is made between personality characteristics that are regarded as relatively stable over time, i.e., personality traits or style, and personality characteristics that are more amenable to change, i.e., characteristics adaptations, (e.g., [5, 6]). In the personality disorder field, characteristic adaptations are often referred to as personality functioning, and include, among others, values, goals, self-concepts and mental representations of others. For the development of an effective treatment plan, it is highly useful for a clinician to gain insight into a patient’s personality aspects that are both maladaptive and changeable.

The Severity Indices of Personality Problems 118 (SIPP-118) is a self-report questionnaire that was specifically designed to measure interpersonal differences in (mal)adaptive personality capacities [5]. The SIPP-118 encompasses 16 facets derived from consensus meetings involving 10 experts in the field of personality pathology. Furthermore, five higher-order factors were proposed based on exploratory factor analyses: social concordance, relational functioning, self-control, responsibility and identity integration^1^. As reported by Pedersen et al. [8], a number of studies have supported clinical relevance, utility, and the relationship between SIPP-118 scores and personality disorder (PD) severity levels [9–16]. However, no consensus has yet emerged as to which scores are best to report: the facet or the higher-order factor scores. Whereas the 16 facets were based on theory and expert opinion, and tested using confirmatory factor analyses, the higher-order structure suggested by the developers was based on exploratory analyses only. This has caused some authors to be more cautious in adopting the higher-order factors, which have moreover been proven difficult to replicate [8].

The facets were developed using an approach that was content-driven: Experts identified concepts, generated items, and these were in turn evaluated by patients. The facets that were included in the instrument showed Cronbach’s alpha values of at least .70 and were found to fit single-factor models well [5]. In a subsequent study conducted by members of the same research group, the psychometric properties of the SIPP-118 were evaluated in two adolescent samples: a patient and non-patient sample [13]. Cronbach’s alpha estimates ranged between .59-.89, with the lowest values being found for the facet respect and the highest for self-respect. Known-groups validity was supported by the finding that a higher degree of pathology as measured by the SIPP-118 was found in the patient sample compared to the non-patient sample. Correlations among facets pertaining to the same higher-order factor varied between .24 and .73, and between .10 and .68 for facets not pertaining to the same higher-order factor. These findings do not provide a clear support for the suggested higher-order factors. All facets except for enduring relationships and responsible industry were sensitive to change in the adolescent patients studied. The largest effect was found for stable self-image. In a recent study, using both a community and two clinical samples, Cronbach’s alpha estimates ranged between .63-.85 (lowest value for the facet respect, highest for aggression regulation and self-respect), with most values exceeding .70 [8]. The authors were not able to replicate the higher-order factors proposed by Andrea and colleagues. The focus of this study is on the facets, since they have a more solid foundation compared to the higher-order factors.

Notably, the SIPP-118 was used in the early stages in the development phase of the diagnostic content for the Levels of Personality Functioning Scale (Criterion A) of the Alternative Model for Personality Disorders [17], especially, with respect to the fine-tuning of severity level descriptions. Furthermore, the SIPP-118 is sometimes used in research studies to obtain an estimate of personality dysfunction; for instance, Bastiaansen and colleagues [18] extracted a single higher-order factor using the SIPP-118, which they used in subsequent analyses to investigate the relationship between personality functioning and personality traits. From a research perspective, it may be useful to obtain one or multiple summary factors for the SIPP facets, (in research, the SIPP is often used as an overall indicator of personality functioning). From a clinical viewpoint, however, using such factor solutions may be suboptimal, since they are mostly based on small samples and exploratory analyses, with the purpose of data reduction rather than obtaining clinically meaningful latent traits. Often, test developers suggest both total and subscale scores to be calculated for their instruments. This type of approach has been criticized by some; if the subscales do not explain substantial portions of variance, it may be more suitable to focus on a total score only [19]. Others have argued that ignoring subscales can lead to an impoverished measurement practice, where crucial characteristics of the patient are overlooked (e.g., [20]).

In this study, we will assess the incremental value of subscale (i.e., facet) scores over and above the total score. We will do so in two steps. First, we will evaluate to what degree the SIPP items tap into an overall general factor (also referred to as a g-PD factor) using bi-factor modeling. Second, we will study the distinctiveness of the facets using proportional reduction in mean squared error (PRMSE) based statistics. We choose to focus on the facets, and not the higher-order factors, since the former have a strong theoretical basis.

## Methods

### Participants

Three large clinical samples were available for the secondary data analysis applied in this study. All patients included in the current study reported symptoms indicative of personality pathology. The data were gathered in different treatment units and subsequently registered in an anonymous central database.

### Norwegian sample

This sample comprised data from 3577 patients consecutively admitted to 17 different treatment units participating in the Norwegian Network of Personality-Focused Treatment Programs [21] between July 2009 and April 2019. The majority of the patients were female (76%), and mean age was 31 years (SD = 9, range 16–64). Most patients in this sample (71%) had a PD. The most common PD was avoidant PD (33%), followed by borderline personality disorder (28%) and PD not otherwise specified (13%). Current major depression was the most common symptom disorder (45%). Further details regarding sociodemographic and diagnostic characteristics have previously been reported by Pedersen and Karterud [22]. The different treatment units collected patient data, which were registered in an anonymous central database, administrated by the Department for Personality Psychiatry, Oslo University Hospital, in Oslo. The State Data Inspectorate and the Regional Committee for Medical Research and Ethics have approved these procedures.

### Dutch samples

The first Dutch sample (henceforth labeled Dutch sample 1) consisted of 4751 patients admitted to specialized care programs for the treatment of PDs from Pro Persona, Mental Health Care, in The Netherlands. Of these, 70% were female, and the mean age was 35 years (SD = 11, range 18–65). Data were collected as part of a Routine Outcome Monitoring procedure between March 2012 and January 2019. Only information about the primary diagnoses was available. Most patients in this sample (75%) had a PD as the primary diagnosis. The most common PD was PD not otherwise specified (37%), followed by borderline PD (17%) and avoidant PD (13%). Unipolar depressive disorder was the most common primary symptom disorder (8%).

The second Dutch sample (henceforth labeled Dutch sample 2) comprised data from 2217 patients who were referred to De Viersprong, a specialized mental health facility for the assessment and treatment of PDs. Data were collected as part of the admission procedure between January 2012 and December 2016. Most patients were female (66%), with an average age of 34 (SD = 11; range 18–67 years). The patient population that was referred to De Viersprong is described in more detail by Weekers et al. [23], and Hutsebaut et al. [24].

### Measures

The SIPP-118 is a self-report questionnaire developed by Andrea et al. [5] that aims to measure core components of (mal)adaptive personality functioning that can change over time. The instrument contains 118 items that cover 16 facets: emotion regulation, aggression regulation, effortful control, frustration tolerance, self-respect, stable self-image, self-reflexive functioning, enjoyment, purposefulness, responsible industry, trustworthiness, intimacy, enduring relationships, feeling recognized, cooperation, and respect. The response categories range from 1 to 4 (fully disagree to fully agree), with higher total scores indicating more adaptive functioning. A recall period of 3 months has been used. The original Dutch version was used in the Dutch samples, and an official Norwegian translation was used in the Norwegian sample.

### Psychometric analyses

In this study we used confirmatory bi-factor analysis [25–27] to establish whether there is a dominant general factor underlying the item responses. The main distinguishing feature of the bi-factor model is that the items load on both the general factor and the so-called group factors. In constrast, in a correlated-trait model, items load on their own respective factors and these factors are allowed to correlate. We refer the interested reader to the online supplement accompanying the paper by Paap et al. [28] for a more detailed comparison of bi-factor analysis to other commonly used techniques for assessing dimensionality. Due to its unique features, a bi-factor model is very well suited to investigate to what degree item variance is attributable to a general factor and/or to specific group factors .

An unconstrained model (loadings and thresholds were allowed to vary across the samples) was estimated. We then calculated the percentage of explained common variance (ECV) that was attributable to the general factor and to group factors (i.e., facets) for each sample separately. The ECV equals the sum of squared factor loadings for the respective factor divided by the sum of all squared factor loadings (the common variance) for the model. Reise et al. [29] tentatively proposed that when the ECV for the general factor in a bi-factor model is larger than 60%, the factor loading estimates for a unidimensional model are close to the true loadings on the general factor in the bi-factor model, and can be interpreted as essentially unidimensional. More recently, O'Connor Quinn [30] proposed a more conservative cut-off of 70%, which was used as a guideline in this study. Model fit was evaluated using the following indices and rules-of-thumb: the comparative fit index (CFI), good fit if CFI ≥ 0.95 and acceptable fit if CFI was between 0.90 and 0.95, and the root mean square error of approximation (RSMEA), good fit if RSMEA ≤ 0.06, acceptable fit if RMSEA was between 0.06 and 0.08 [31, 32].

To ascertain whether the facet scores showed a sufficient degree of distinctiveness (unique information for score interpretation not captured by the total score and other subscores), we used a method proposed by Haberman [33]. Within a classical test theory framework, Haberman [33] outlined that for a subscale score to have added value of being reported, the proportional reduction in mean square error (PRMSE) in the estimate of the true subscale score from the observed subscale score should be larger than the PRMSE from the observed total score. In other words, the observed subscale score should explain more variance in the true subscale score than the observed total score does. This requirement can be expressed as a value-added ratio as introduced by Feinberg and Wainer [34]: VAR = PRMSE(subscale)/PRMSE(total). Feinberg and Jurich [35] provided the following guideline: VAR ≥ 1.1 is indicative of a minimally meaningful added value of the subscale score. Note that the PRMSE(subscale) equals the reliability of the subscale score (i.e., % of explained variance in the subscale true score by the observed subscale score), and that value added can only be achieved for subscales that are reliable and to some extent distinct from other subscale scores. Here, the subscales correspond to the facets. The value-added ratios were calculated for all 16 facets, separately for each of the three samples. Non-parametric bootstrapping with 2500 resamples was used to calculate quantile-based confidence intervals for the facet VAR statistics and to assess pairwise differences in VAR between facets.

### Software

All statistical analyses were performed in the open source software program R version 3.4.3 [36]. Custom coding was used for the PRMSE-based analyses. The bi-factor model was estimated using the bfactor function in the R package mirt version 1.30 [37], which is based on a full information maximum likelihood approach and follows the analytic strategy outlined by Cai [25]. The SIPP-118 likert-type items were treated as proper polytomous responses in a bi-factor model estimated under the item response theory (IRT) paradigm using full information maximum likelihood with an Expectation-Maximization algorithm. In contrast to confirmatory factor analysis (CFA) that only uses limited-information statistics such as covariances and means, IRT makes use of the full item response patterns. As both paradigms are latent variable models, the IRT model parameters can be readily reexpressed in traditional factor analysis loadings and thresholds to report in a metric that is familiar to most readers.

## Results

### Descriptive statistics

Table 1 shows mean scores for the subscales/facets across all samples. Small differences in mean facet scores were found across the groups (0.00-0.23, with a mean of 0.06, and only nine observed differences above 0.10). The magnitude of these differences was not regarded as clinically significant.

**Table 1:** Mean scores (standard deviations) for the subscales/facets

Facet no.	Scale/facet name	Norweg N=3577	Dutch 1 N=4751	Dutch 2 N=2217
		Mean (SD)	Mean (SD)	Mean (SD)
1	Emotion regulation	2.43 (0.71)	2.47 (0.68)	2.40 (0.71)
2	Effortful control	2.58 (0.74)	2.61 (0.68)	2.56 (0.75)
3	Self-respect	2.15 (0.69)	2.15 (0.66)	2.20 (0.68)
4	Stable self-image	2.50 (0.67)	2.33 (0.64)	2.26 (0.67)
5	Self-reflective functioning	2.49 (0.64)	2.41 (0.64)	2.36 (0.66)
6	Enjoyment	2.35 (0.64)	2.28 (0.63)	2.33 (0.66)
7	Purposefulness	2.45 (0.65)	2.40 (0.65)	2.40 (0.66)
8	Intimacy	2.61 (0.67)	2.49 (0.69)	2.50 (0.70)
9	Enduring relationships	2.56 (0.63)	2.41 (0.64)	2.41 (0.66)
10	Feeling recognized	2.54 (0.61)	2.51 (0.60)	2.51 (0.59)
11	Responsible industry	2.77 (0.65)	2.87 (0.63)	2.80 (0.66)
12	Trustworthiness	3.06 (0.59)	3.10 (0.57)	3.03 (0.63)
13	Aggression regulation	3.20 (0.69)	3.29 (0.70)	3.26 (0.73)
14	Frustration tolerance	2.22 (0.54)	2.26 (0.57)	2.24 (0.57)
15	Cooperation	2.89 (0.60)	2.82 (0.59)	2.75 (0.61)
16	Respect	3.23 (0.53)	3.16 (0.52)	3.10 (0.56)

*Norweg* norwegian sample, *Dutch 1* dutch sample 1, *Dutch 2* dutch sample 2

### Bi-factor analyses

The estimated model showed adequate to good fit in terms of RMSEA (.03), and CFI (.93). The ECV attributable to the general factor was virtually identical across the groups, ranging from 49.6 to 50.3 (see Table 2). An ECV of 50% cannot be considered high enough to warrant treating the instrument as unidimensional. Although the general factor explained a sizeable amount of variance, so did the facets.

**Table 2:** Percentage of explained common variance for the estimated bi-factor model

	Norweg	Dutch 1	Dutch 2
	*ECV*	*ECV*	*ECV*
General factor	50.1	50.3	49.6
Emotion regulation	1.6	2.1	1.6
Effortful control	1.6	2.1	1.8
Self-respect	5.3	4.8	5.2
Stable self-image	1.3	1.6	2.0
Self-reflective functioning	2.2	2.1	2.3
Enjoyment	4.2	3.3	3.9
Purposefulness	3.6	3.3	3.6
Intimacy	5.2	4.3	4.7
Enduring relationships	3.3	3.0	3.1
Feeling recognized	3.2	2.6	2.6
Responsible industry	2.8	3.1	3.1
Trustworthiness	3.3	3.8	4.0
Aggression regulation	3.5	5.0	4.2
Frustration tolerance	1.8	2.1	1.8
Cooperation	4.0	3.3	3.5
Respect	2.9	3.2	3.0

*Norweg* norwegian sample, *Dutch 1* dutch sample 1, *Dutch 2* dutch sample 2

### Distinctiveness of the facets

Almost all facets had a VAR ≥ 1.1 (Table 3). The exceptions were stable self-image (all three samples), and frustration tolerance (Norwegian and Dutch sample 2). Note that the VAR value for frustration tolerance only just exceeded 1.1 for Dutch sample 1. The VAR values showed considerable variation, ranging between 0.97 (stable self-image, Dutch sample 1) and 2.96 (intimacy, Norwegian sample). The facets with the largest effect sizes across all samples were intimacy and trustworthiness. Also in the top five of highest VAR values across all samples were aggression regulation and self-respect.

**Table 3:** Distinctiveness of the SIPP-118 facets

Facet no.	Norweg			Dutch 1			Dutch 2		
	PRMSE(s)	PRMSE(x)	VAR	PRMSE(s)	PRMSE(x)	VAR	PRMSE(s)	PRMSE(x)	VAR
1	0.82	0.64	**1.28**	0.82	0.63	**1.31**	0.83	0.65	**1.28**
2	0.82	0.59	**1.39**	0.80	0.59	**1.35**	0.83	0.61	**1.37**
3	0.83	0.48	**1.74**	0.83	0.45	**1.85**	0.83	0.43	**1.92**
4	0.78	0.74	1.06	0.77	0.79	0.97	0.79	0.78	1.01
5	0.76	0.62	**1.22**	0.78	0.62	**1.27**	0.78	0.60	**1.30**
6	0.78	0.46	**1.69**	0.78	0.49	**1.60**	0.80	0.44	**1.82**
7	0.75	0.64	**1.18**	0.78	0.62	**1.25**	0.76	0.60	**1.26**
8	0.80	0.27	**2.96**	0.82	0.40	**2.04**	0.82	0.34	**2.42**
9	0.73	0.51	**1.44**	0.77	0.56	**1.39**	0.77	0.58	**1.34**
10	0.77	0.62	**1.25**	0.79	0.66	**1.19**	0.76	0.67	**1.14**
11	0.75	0.50	**1.49**	0.75	0.47	**1.60**	0.77	0.47	**1.63**
12	0.77	0.49	**1.56**	0.77	0.42	**1.85**	0.80	0.40	**1.98**
13	0.86	0.44	**1.95**	0.89	0.42	**2.13**	0.88	0.44	**2.00**
14	0.75	0.69	1.09	0.77	0.69	**1.12**	0.76	0.71	1.07
15	0.78	0.51	**1.54**	0.79	0.55	**1.44**	0.80	0.53	**1.51**
16	0.72	0.47	**1.55**	0.73	0.41	**1.77**	0.75	0.47	**1.58**

*PRMSE* proportional reduction in mean squared error, *s* subscale, *x* total score, *VAR* value-added ratio, *Norweg* norwegian sample, *Dutch 1* dutch sample 1, *Dutch 2* dutch sample 2, *VAR* values ≥ 1.1 are printed in bold

**Fig. 1:**
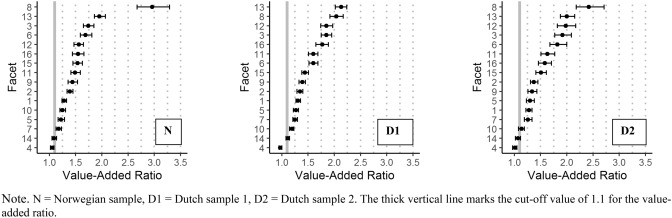
Value-added ratio (x-axis) with confidence interval for each of the 16 SIPP-118 facets (y-axis). *N* norwegian sample, *D1* dutch sample 1, *D2* dutch sample 2. The thick vertical line marks the cut-off value of 1.1 for the value-added ratio

Figure 1 shows the value-added ratios alongside their confidence intervals, the facets are ordered based on their VAR to ease interpretation. When refining the cut-off rule such that the confidence interval around a VAR value should not include 1.1 (i.e., the VAR value should differ significantly from 1.1), this criterion was not met for the following facets: stable self-image (all samples), frustration tolerance (all samples), and feeling recognized (Dutch sample 2).

## Discussion

In this study, we focused on evaluating the relative strength of the 16 facets of the SIPP-118. Having 16 facets at one’s disposal allows for a detailed picture of patients’ adaptive and maladaptive capacities, but results in a number of scores that might be overwhelming to interpret in daily clinical practice. The question arises, therefore, whether it is worth the trouble to both patient and clinician to obtain and interpret all 16 facet scores. Our results indicate that 14 out of 16 facets have a clear incremental value. Moreover, the general factor that we extracted in our bi-factor analyses was not strong enough to warrant using the SIPP-118 as a unidimensional measure. The outcomes were highly similar across the large clinical Dutch and Norwegian samples we used, supporting generalizability of our findings.

In recent decades, there has been a strong call for moving from a categorical to a dimensional approach to PD diagnoses, (e.g., [38, 39]). As an effect, there has been an increased interest in the so-called p-factor (general factor of psychopathology, e.g., [40]) or g-PD factor (general factor of PD, e.g., [41, 42]). A number of previous studies using interview-rated PD criteria have found a strong relationship between borderline PD traits and the g-PD [41, 43]. In our study, we did not find a strong general factor. This may be partly due to the content of the SIPP-118, which was designed to assess changeable aspects of maladaptive personality functioning, and the items do not necessarily directly reflect the different DSM-5 PDs. Furthermore, multidimensionality was explicitly introduced during the item generation phase.

Previous studies have yielded inconsistent findings with respect to the higher-order factor structure of the SIPP-118 (see [8]). It is unclear what caused these inconsistencies, but since this higher-order structure was informed by exploratory factor analysis only, it may not be surprising that the results differ across studies. Exploratory analyses may be particularly sensitive to sample characteristics, and not generalize well. In this study, we used an analytic approach with a specific focus on the facets. The results were observed to be comparable across the three samples. Although more research is needed to ascertain whether the generalizability holds for different subgroups and non-European countries, the results so far are reassuring. Overall, we found strong support for the facets. That being said, the facets stable self-image and frustration tolerance did not show sufficient distinctiveness (the VAR values for these facets did not differ significantly from 1.1, this was true for all samples). As described by Feinberg and Jurich [35], the goal of reporting subscores is to allow for fine-grained inferences from the item responses. However, reporting subscores that do not have a demonstrated added value may result in decisions being made based on misinformation and incorrect representations of the trait being measured. We suggest the facets stable self-image and frustration tolerance be used with caution or not at all.

The SIPP is a valuable instrument that is not tied to a particular model of PD. We would like to stress that we do not suggest that solely the SIPP be used in diagnosis. The patient perspective is important and should be central in certain situations, but it does not paint the whole picture. It has been repeatedly shown that self-report instruments cannot be used as a proxy for (or replacement of) clinical diagnosis (see [44]). This may be especially true for certain types of PDs, such antisocial PD [45]. As to the question posed in the title of this article, our results suggest that yes—we really do need those facets! If it is not feasible in a given situation to administer the entire instrument, one possibility would be to make a selection of facets, depending on the goal for which the instrument is being used. For obtaining a general severity score, we would suggest to use an instrument that shows a stronger g-PD factor.

## Footnotes

1 The computation was based on primary and secondary factor loadings as regression weights for the facets combined with clinical judgment [7]. Note that some facets are thought to load on multiple higher-order factors.
